# sRNA expression profile of KPC-2-producing carbapenem-resistant *Klebsiella pneumoniae*: Functional role of sRNA51

**DOI:** 10.1371/journal.ppat.1012187

**Published:** 2024-05-08

**Authors:** Yibo Bai, Chonghong Xie, Yue Zhang, Zhijie Zhang, Jianhua Liu, Guixue Cheng, Yan Li, Di Wang, Bing Cui, Yong Liu, Xiaosong Qin

**Affiliations:** 1 Department of Laboratory Medicine, Shengjing Hospital of China Medical University, Shenyang, Liaoning Province, China; 2 Liaoning Clinical Research Center for Laboratory Medicine, Shenyang, Liaoning Province, China; Catalan Institute for Water Research (ICRA), SPAIN

## Abstract

The emergence of carbapenem-resistant *Klebsiella pneumoniae* (CRKP) has significant challenges to human health and clinical treatment, with KPC-2-producing CRKP being the predominant epidemic strain. Therefore, there is an urgent need to identify new therapeutic targets and strategies. Non-coding small RNA (sRNA) is a post-transcriptional regulator of genes involved in important biological processes in bacteria and represents an emerging therapeutic strategy for antibiotic-resistant bacteria. In this study, we analyzed the transcription profile of KPC-2-producing CRKP using RNA-seq. Of the 4693 known genes detected, the expression of 307 genes was significantly different from that of carbapenem-sensitive *Klebsiella pneumoniae* (CSKP), including 133 up-regulated and 174 down-regulated genes. Both the Kyoto Encyclopedia of Genes and Genomes (KEGG) pathway enrichment and Gene Ontology (GO) analysis showed that these differentially expressed genes (DEGs) were mainly related to metabolism. In addition, we identified the sRNA expression profile of KPC-2-producing CRKP for the first time and detected 115 sRNAs, including 112 newly discovered sRNAs. Compared to CSKP, 43 sRNAs were differentially expressed in KPC-2-producing CRKP, including 39 up-regulated and 4 down-regulated sRNAs. We chose sRNA51, the most significantly differentially expressed sRNA in KPC-2-producing CRKP, as our research subject. By constructing sRNA51-overexpressing KPC-2-producing CRKP strains, we found that sRNA51 overexpression down-regulated the expression of *acrA* and alleviated resistance to meropenem and ertapenem in KPC-2-producing CRKP, while overexpression of *acrA* in sRNA51-overexpressing strains restored the reduction of resistance. Therefore, we speculated that sRNA51 could affect the resistance of KPC-2-producing CRKP by inhibiting *acrA* expression and affecting the formation of efflux pumps. This provides a new approach for developing antibiotic adjuvants to restore the sensitivity of CRKP.

## Author summary

The widespread prevalence of KPC-2-producing CRKP presents a significant challenge to its clinical treatment. Studies identified sRNA-mediated regulation as a potential viable RNA-based drug target against antibiotic-resistant bacteria. However, little is known regarding the expression profile and functions of sRNAs in KPC-2-producing CRKP. In our study, we revealed the sRNA expression profile of KPC-2-producing CRKP and identified 112 new sRNAs. Through the overexpression of sRNA51 or *acrA* in KPC-2-producing CRKP, we established that sRNA51 could regulate the resistance of KPC-2-producing CRKP by inhibiting the expression of *acrA*. This provides a new target for the clinical treatment of KPC-2-producing CRKP at the sRNA level.

## Introduction

*Klebsiella pneumoniae* is one of the major pathogens causing nosocomial infections, and its antimicrobial resistance has received a lot of attention [1]. Carbapenem-resistant *Klebsiella pneumoniae* (CRKP) is recognized as an urgent public health threat by the Centers for Disease Control and Prevention (CDC) [2,3]. CRKP is resistant to almost all classes of common antimicrobial drugs, including imipenem, meropenem, and ertapenem [4]. It is also an important factor contributing to increasing infection-related morbidity and mortality, with blood infections having a mortality rate as high as approximately 50%, posing a substantial challenge to clinical anti-infective treatment [2].

Carbapenemase synthesis is one of the most important resistance mechanisms in CRKP [5]. According to the Ambler classification, common carbapenemases can be divided into three classes: Class A (KPC and IMI), Class B (NDM, IMP, and VIM), and Class D (OXA) [5]. Among these, KPC-2-producing CRKP is the most prevalent globally and in China [6–8]. Consistent with other studies, out of 153 CRKP strains isolated from patient specimens in our hospital from 2019 to 2021, 121 (79.1%) were found to produce KPC-2 (S1 Table).

For decades, researchers have been devoting to develop effective method to treat KPC-2-producing CRKP infections, and polymyxin and tigecycline were considered the last resort against it [9]. However, adverse reactions, such as organ toxicity, and the problem of plasma drug concentrations have limited their clinical application [5]. Additionally, increasing reports of CRKP resistance to polymyxin and tigacycline pose a major challenge for healthcare [10,11]. The rapid evolution of antimicrobial resistance in KPC-2-producing CRKP and the slow development of new antibiotics have driven the search for approaches to re-sensitize resistant strains to existing antibiotics; for example, ceftazidime/avibactam was approved in 2015 in the USA [12]. Avibactam is a novel synthetic β-lactamase inhibitor that can restore bacterial sensitivity to cephalosporins by inhibiting KPC carbapenemase [13]. Although ceftazidime/avibactam-based therapies have shown relatively good clinical efficacy in the treatment of KPC-2-producing CRKP infections, an increasing number of avibactam-resistant *K*. *pneumoniae* isolates have been reported [14,15]. Therefore, novel therapeutic targets and strategies need to be identified.

Currently, RNA-based therapies are a promising strategy considering that bacteria can regulate their properties, including antibiotic resistance, by regulating RNA expression [16]. Na et al. designed several synthetic non-coding small RNAs (sRNAs) targeting different mRNAs that regulated gene expression in different *Escherichia coli* strains [17]. Since then, several studies have explored the application of synthetic sRNAs in metabolic engineering and synthetic biology [18–20]. sRNAs, ranging from 50–500 nt in bacteria, do not encode proteins but can inhibit or promote translation of mRNA by complementing with their target mRNAs. Thus, sRNA is a post-transcriptional regulator of genes involved in important cellular processes, such as bacterial growth, virulence, and drug resistance, and is considered an RNA-based drug target [21,22]. For instance, sRNA00203 is known to induce antibiotic resistance in *Acinetobacter baumannii* by promoting biofilm formation [23]. Additionally, sRNA1039 regulates *Shigella sonnei* sensitivity to ampicillin, gentamicin, and cefuroxime by regulating the expression of the target gene *CFA* mRNA [24]. Therefore, it is important to explore the expression profile and functions of sRNAs in KPC-2-producing CRKP.

AcrA is a component of the multidrug efflux pump AcrAB-TolC, which acts as a membrane fusion protein that assembles the inner and outer membrane components of the pump [25]. It has been shown to collaborate with other mechanisms, conferring bacteria high levels of resistance to multiple drugs, including carbapenems [26]. Several regulatory mechanisms are involved in *acrA* expression in *E*. *coli*, including the global regulators *MarA*, *CsrA*, and *RamA* [27]. There is increasing evidence that supports the relationship between the upregulation of *acrA* expression and CRKP resistance; however, the mechanism of *acrA* up-regulation in CRKP remains unknown [28,29].

We aimed to mine the sRNA expression profile of KPC-2-producing CRKP using RNA-seq and analyze the function of sRNA51, the most differentially expressed sRNA, in the resistance of KPC-2-producing CRKP. We identified 115 sRNAs in KPC-2-producing CRKP, including 112 newly discovered sRNAs. Compared to CSKP, 43 sRNAs were differentially expressed in KPC-2-producing CRKP, including 39 up-regulated and 4 down-regulated sRNAs. Using biological information software and interfering with the expression of sRNA51 in KPC-2-producing CRKP, we demonstrated that sRNA51 could regulate carbapenems resistance in KPC-2-producing CRKP by inhibiting *acrA* expression. Therefore, sRNA51 could serve as a potential new target for the clinical treatment of KPC-2-producing CRKP. This also provides new idea for developing antibiotic adjuvants to restore the drug sensitivity of drug-resistant pathogens.

## Results

### Validation of resistance of KPC-2-producing CRKP and CSKP strains to common antibiotics including carbapenems

We randomly selected five strains of KPC-2-producing CRKP and five strains of CSKP using the random sampling method in Statistical Product Service Solutions (SPSS) from the pathogen bank of Shengjing Hospital affiliated to China Medical University, as shown in S2 Table. First, we used the Phoneix 100 system to detect the resistance of ATCC 700603 (standard CSKP strain), ATCC 1705 (standard KPC-2-producing CRKP strain), and the above-mentioned 10 clinical isolates. The KPC-2-producing CRKP were resistant to carbapenems and other drugs such as cefepime, amikacin, and amtraxam, consistent with previous studies (S2 Table) [30]. In order to verify the resistance and resistance level of the strains to carbapenems, we further determined the minimal inhibitory concentrations (MIC) of meropenem, ertapenem, and imipenem to them, and the results are shown in Table 1.

**Table 1:** Minimal inhibitory concentrations of carbapenems for *K*. *pneumoniae*.

MIC	CSKP					KPC-2 CRKP					ATCC 700603	ATCC 1705
	1	2	3	4	5	1	2	3	4	5		
Meropenem (μg/mL)	≤1	≤1	≤1	≤1	≤1	64	64	128	64	64	≤1	64
Ertapenem(μg/mL)	≤1	≤1	≤1	≤1	≤1	512	512	512	256	512	≤1	512
Imipenem (μg/mL)	≤1	≤1	≤1	≤1	≤1	>1024	>1024	>1024	>1024	>1024	≤1	>1024

### Mining of the transcription profile and DEGs of KPC-2-producing CRKP using RNA-seq

**Fig 1:**
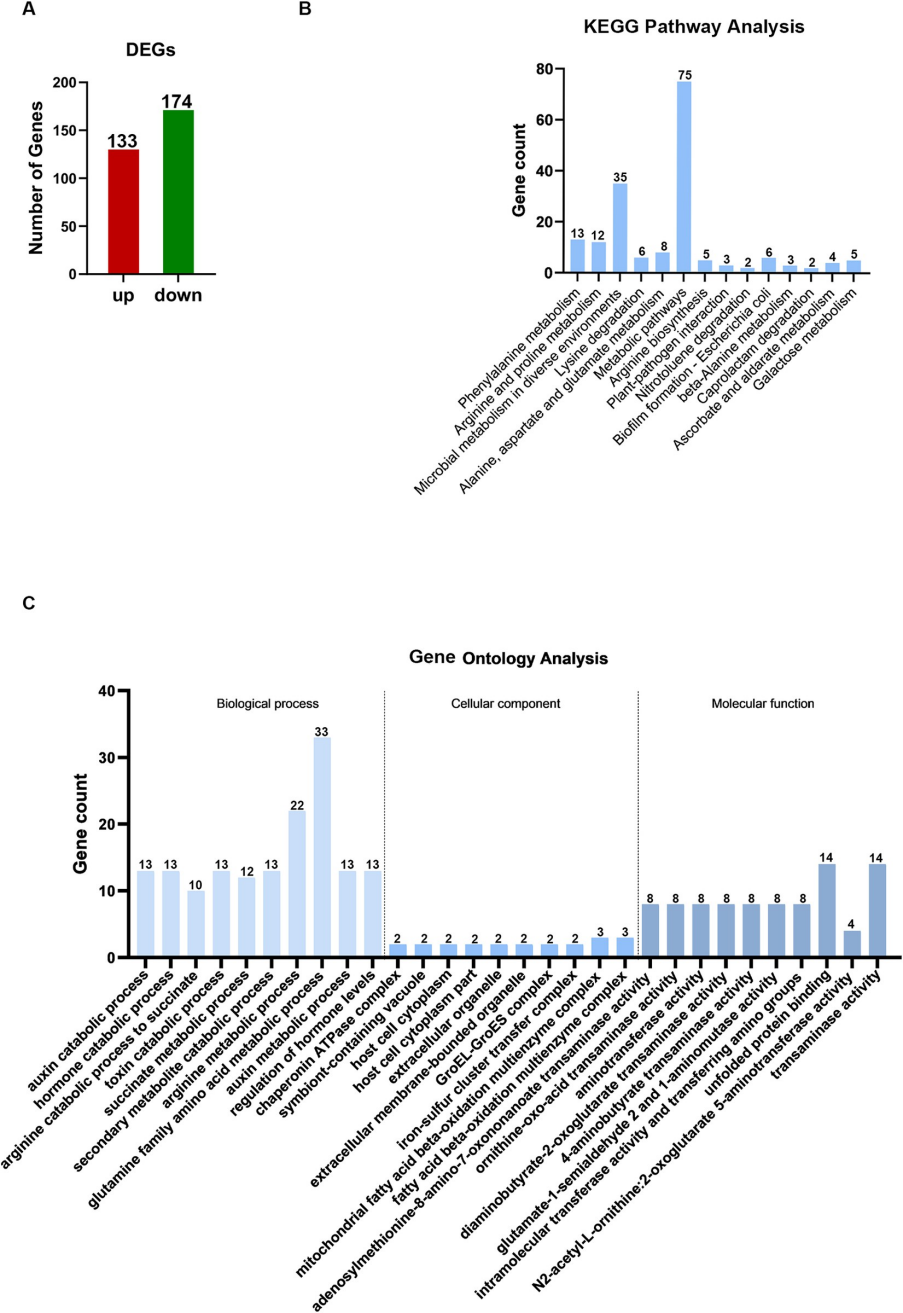
Analysis of RNA-seq data. (A) FDR and log2FC were used to screen for DEGs. (B) KEGG pathway enrichment analysis of DEGs. Fourteen significant enriched pathways (*P* < 0.05) are represented. (C) GO analysis of DEGs. DEGs are divided into three categories: biological processes, molecular functions, and cellular components. The Y-axis represents the number of genes corresponding to each classification entry and the X-axis represents meaningful enrichment pathways (*P* < 0.05).

We used above five KPC-2 producing CRKP strains and five CSKP strains for RNA-seq to mine the transcription profile and differentially expressed genes (DEGs) of KPC-2 producing CRKP. After aligning the RNA-seq readings to the reference genome assembly GCF022869665.1, approximately 88–95% of the reads were mapped. A total of 4693 known genes in KPC-2 producing CRKP and CSKP were obtained. Data generated were deposited in the Sequence Read Archive (SRA) at the National Center for Biotechnology Institute (accession no. PRJNA1022581). Subsequently, EdgeR software was employed to screen DEGs using the criteria of False Discovery Rate (FDR) < 0.05, and | log2FC | > 1. The results showed that 307 genes were differentially expressed in KPC-2 producing CRKP compared to CSKP, including 133 up-regulated and 174 down-regulated genes (Fig 1A and S3 Table).

Additionally, Kyoto Encyclopedia of Genes and Genomes (KEGG) pathway enrichment and Gene Ontology (GO) analysis were performed to further understand the functions of these DEGs. KEGG pathway enrichment analysis showed that the DEGs were mapped to 77 pathways (S4 Table). Fourteen pathways were significantly enriched (*P* < 0.05) of which 12 were related to metabolism, including phenylalanine metabolism, arginine and proline metabolism, and alanine, aspartate, and glutamate metabolism (Fig 1B). In addition, GO analysis revealed that the DEGs were significantly enriched (*P* < 0.05) 185 terms in biological processes, 24 terms in cellular components, and 97 terms in molecular functions (S5 Table). Moreover, we exhibited the first 10 significant enrichment terms in each class in Fig 1C, and identified that DEGs were mainly related to energy and metabolism.

### Identification of the sRNA expression profile and differentially expressed sRNAs of KPC-2-producing CRKP

As described in previous studies [31, 32], we analyzed the length and secondary structure of genes that could not be aligned with the reference genome and Non-Redundant Protein (NR) database, and obtained 115 candidate sRNAs. We annotated candidate sRNAs using the sRNAMap database and Rfam databases, and identified three known sRNAs, while the remaining 112 were newly discovered sRNAs (S6 Table). Using the criteria of FDR < 0.05 and | log2FC | > 1, we obtained 43 differentially expressed sRNAs in KPC-2-producing CRKP, ranging in length from 50 to 500 nt, including 39 up-regulated sRNAs and four down-regulated sRNAs, compared to CSKP (Table 2). Among them, based on the | log2FC | value, sRNA51 was found to be the most significantly differentially expressed sRNA in KPC-2-producing CRKP, with its expression level being almost 16 times lower than that in CSKP.

**Table 2:** Differentially expressed sRNAs of KPC-2-producing CRKP.

Gene	CSKP TPM	KPC-2 TPM	log2FC	*P*-value	FDR	Length(nt)
sRNA51	73.472	0.001	-16.1649	0.0004	0.0013	180
sRNA474	45.494	0.001	-15.4734	0.0029	0.0078	70
sRNA399	191.004	10.478	-4.1882	0.0047	0.0117	51
sRNA156	425.002	35.534	-3.5802	0.0066	0.0147	120
sRNA443	23.9820	382.3900	3.9950	0.0000	0.0000	458
sRNA196	34.6200	403.6200	3.5433	0.0000	0.0000	473
sRNA418	261.5800	2748.7900	3.3935	0.0000	0.0000	95
sRNA56	59.4900	567.7200	3.2545	0.0000	0.0000	328
sRNA219	102.0300	671.7560	2.7189	0.0000	0.0000	60
sRNA211	56.5500	366.6320	2.6967	0.0001	0.0006	320
sRNA330	743.0700	4788.5240	2.6880	0.0000	0.0000	56
sRNA308	119.2780	768.4180	2.6876	0.0000	0.0000	54
sRNA212	83.7020	498.2100	2.5734	0.0000	0.0003	75
sRNA73	43.1960	245.9260	2.5093	0.0000	0.0000	82
sRNA209	51.4660	288.8620	2.4887	0.0000	0.0001	53
sRNA347	17.1040	90.7660	2.4078	0.0001	0.0006	52
sRNA299	45.0120	233.1940	2.3731	0.0000	0.0000	53
sRNA102	217.8840	1074.2660	2.3017	0.0000	0.0000	115
sRNA42	85.7280	379.5500	2.1465	0.0000	0.0000	81
sRNA84	61.8520	223.9820	1.8565	0.0000	0.0000	79
sRNA352	268.3800	926.6620	1.7878	0.0000	0.0000	75
sRNA11	123.3420	420.8100	1.7705	0.0000	0.0001	63
sRNA55	299.5180	1020.6440	1.7688	0.0003	0.0010	183
sRNA380	51.4200	167.1420	1.7007	0.0002	0.0009	50
sRNA303	651.2420	2075.6100	1.6723	0.0000	0.0000	64
sRNA224	76.0020	240.6240	1.6627	0.0003	0.0011	66
sRNA445	87.9700	265.1120	1.5915	0.0001	0.0003	58
sRNA136	86.4880	247.3180	1.5158	0.0000	0.0001	59
sRNA217	195.3540	515.1760	1.3990	0.0002	0.0009	66
sRNA369	246.8160	642.2280	1.3796	0.0000	0.0001	52
sRNA456	131.6560	332.1080	1.3349	0.0003	0.0011	98
sRNA318	497.5020	1245.5580	1.3240	0.0153	0.0309	250
sRNA438	166.2680	412.4140	1.3106	0.0002	0.0008	240
sRNA93	212.5260	521.9280	1.2962	0.0005	0.0016	126
sRNA215	118.2600	290.3080	1.2956	0.0001	0.0005	73
sRNA295	90.1220	206.3320	1.1950	0.0079	0.0169	167
sRNA258	119.5200	272.4760	1.1889	0.0004	0.0015	109
sRNA315	273.6560	619.5980	1.1790	0.0000	0.0001	85
sRNA398	150.2140	337.2680	1.1669	0.0006	0.0020	134
sRNA439	225.7760	495.1920	1.1331	0.0002	0.0010	134
sRNA297	276.1800	589.9880	1.0951	0.0051	0.0121	121
sRNA454	99.5000	211.8360	1.0902	0.0189	0.0374	50
sRNA449	78.5960	157.8820	1.0063	0.0052	0.0121	102

Transcripts per million (TPM) were used to measure the gene or transcript expression. Log2FC represents the ratio of expression between the two groups and was logarithmic, with a base of two. *P*-value indicates the statistical test value, FDR is the false discovery rate and obtained by correcting for the *P*-value; both FDR and *P* < 0.05 represent statistically significant differences. Length (nt) represents the length of the sRNA.

### Verification of differentially expressed sRNAs of KPC-2-producing CRKP

**Fig 2:**
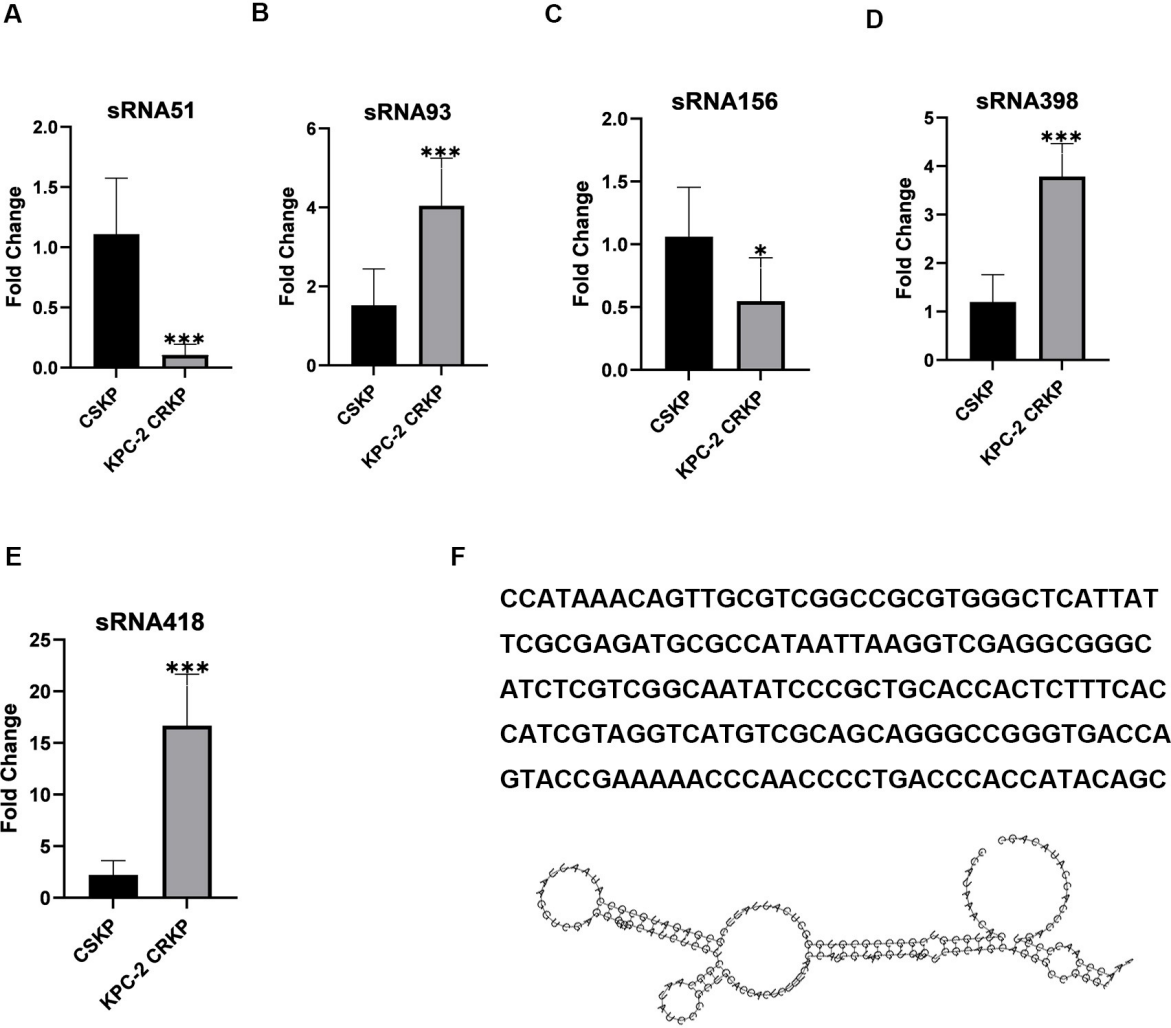
Validation of differentially expressed sRNAs in KPC-2-producing CRKP. The expressions of sRNA51 (A), sRNA93 (B), sRNA156 (C), sRNA398 (D), and sRNA418 (E) in CSKP and KPC-2-producing CRKP were detected by qRT-PCR. Data are expressed as mean ± SD (n = 10), * *P* < 0.05 and *** *P* < 0.001. (F) The secondary structures and gene sequences of sRNA51.

To verify the presence and expression of differentially expressed sRNAs in KPC-2-producing CRKP, we examined the transcript levels of sRNA51 and four other randomly selected differentially expressed sRNAs (sRNA93, sRNA156, sRNA398, and sRNA418, selected by random sampling method of SPSS) using qRT-PCR in our randomly selected additional five KPC-2-producing CRKP strains and five CSKP strains, as well as the above-mentioned 10 strains (the information and resistance to common antibiotics are shown in S7 Table). The qRT-PCR results were consistent with the RNA-seq results (Fig 2A–2E). We also measured the expression levels of these sRNAs in ATCC 1705 and ATCC 700603 strains, and the results are shown in S1 Fig. Subsequently, we selected sRNA51 for further experiments. Secondary structures and gene sequences are shown in Fig 2F. We used CPAT software to detect the presence of Open Reading Frame (ORF) in sRNA51, and the results showed a Fickett score of < 0.74 and a negative hexamer score, which further proved that sRNA51 did not have the ability to encode proteins (Table 3).

**Table 3:** The ORF values of sRNA51.

ID	Start	End	ORF size	Fickett score of KPC-2 CRKP	Hexamer score
sRNA51	43	58	48	0.7004	-2.069333997
sRNA51	119	167	48	0.7004	-2.069333997

ORF size represents the ORF length. Fickett score ≤ 0.74 indicates no ability to encode protein. Fickett score ≥ 0.95 indicates the ability to encode protein. Fickett score between 0.74 and 0.95 indicates uncertainty about the coding. The higher the Hexamer fraction, the stronger its ability to encode proteins.

### Prediction and validation of the target mRNA of sRNA51

**Fig 3:**
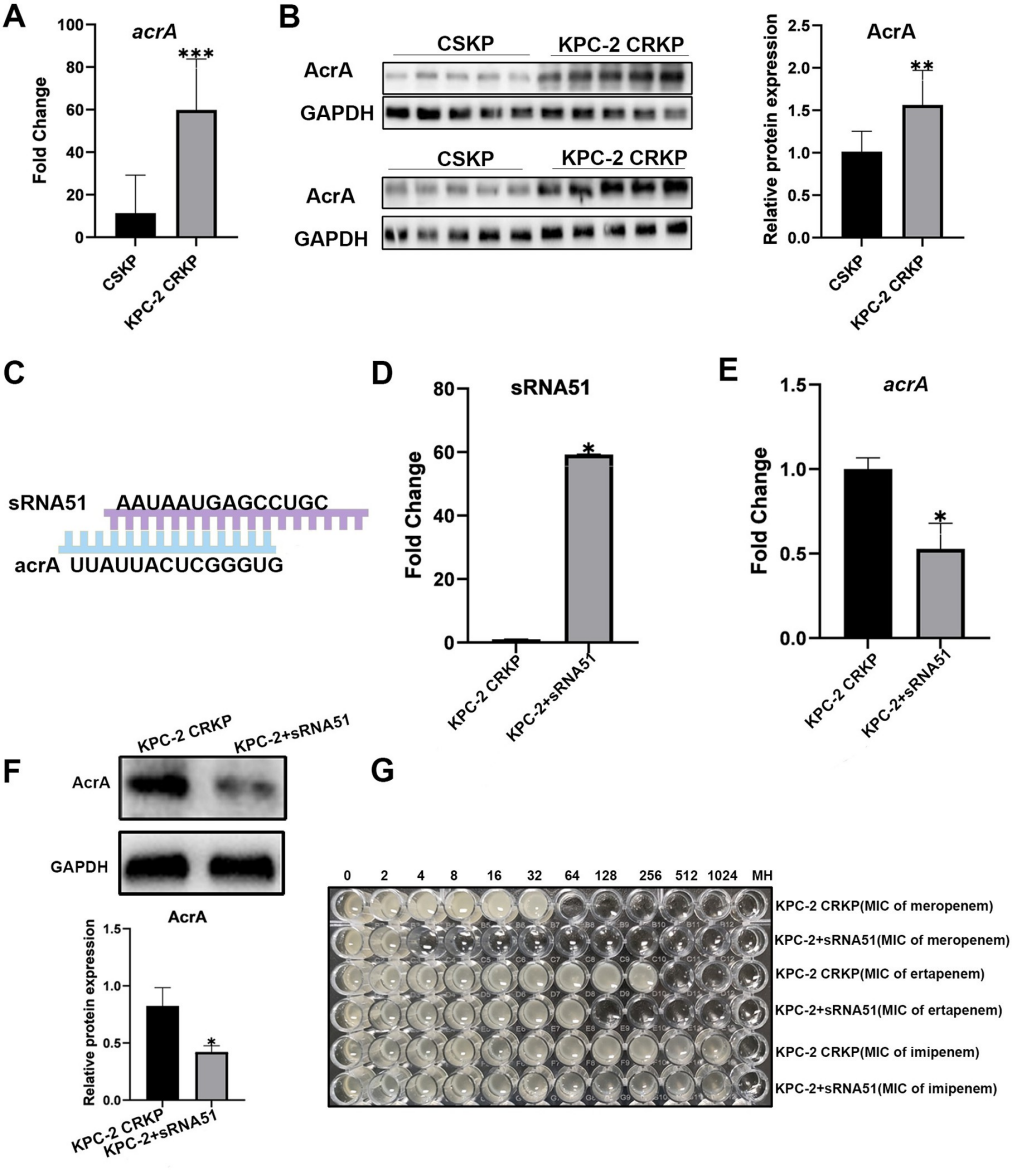
Validation of the target mRNA of sRNA51. The RNA (A) and protein (B) expression level of acrA in CSKP and KPC-2-producing CRKP were detected by qRT-PCR and western blotting (n = 10). (C) A schematic of the binding of sRNA51 and *acrA*. The RNA expression levels of sRNA51 (D) and *acrA* (E) in KPC-2-producing CRKP and KPC-2+sRNA51 strains were detected by qRT-PCR (n = 3). (F) Western blotting analysis of the protein expression level of acrA in KPC-2-producing CRKP and KPC-2+sRNA51 strains. The data presented are representative images of three independent experiments with similar results (S3 Fig). (G) Resistance of KPC-2 producing CRKP and KPC-2+sRNA51 strains to meropenem, ertapenem and imipenem was detected by microbroth dilution. Data are expressed as mean ± SD, * *P* < 0.05, **P < 0.01 and ***P < 0.001.

To further understand the role of sRNA51, we used IntaRNA 2.0.3 to predict its target mRNA. The results included several mRNAs such as *yaiY*, *gabT*, *msrA*, *acrA*, *insA1*, and *holE* (S8 Table), where the expression of *insA1* and *gabT* was reduced, while that of *yaiY*, *msrA*, *acrA*, and *holE* was increased. Studies have shown that acrA, a membrane fusion protein, binds AcrB and TolC to form efflux pumps, and its increased expression is an important cause of carbapenems resistance in gram-negative pathogens, including CRKP [25,33]. Using qRT-PCR and western blotting, we found that the RNA and protein expression levels of acrA in KPC-2-producing CRKP were significantly higher than those in CSKP, which was consistent with our RNA-seq results and previous studies [28,34] (Fig 3A and 3B). We further predicted that sRNA51 would bind to the coding region of *acrA* through IntaRNA 2.0.3, the binding schematic diagram is shown in Fig 3C. Therefore, we hypothesized that sRNA51 could bind to *acrA* and inhibit its expression, thus affecting carbapenems resistance in *K*. *pneumoniae*.

To confirm the function of sRNA51 in KPC-2-producing CRKP, we generated sRNA51-overexpressing KPC-2-producing CRKP strains (KPC-2+sRNA51). qRT-PCR and western blotting results showed that the RNA and protein expression levels of acrA in the KPC-2+sRNA51 strains were lower than those in the KPC-2-producing CRKP (Fig 3D–3F). Considering that the efflux pumps can affect the resistance of pathogens to antimicrobial agents, we tested the resistance of the KPC-2+sRNA51 strains. Compared with KPC-2-producing CRKP, resistance to meropenem was reduced from 64 μg/mL to 4 μg/mL and ertapenem from 512 μg/mL to 128 μg/mL in KPC-2+sRNA51 strains, but resistance to imipenem was not significantly changed (Fig 3G and Table 4). This suggests that sRNA51 affects the production of efflux pumps by binding to *acrA*, thereby influencing the resistance of KPC-2-producing CRKP. The above experiments were repeated using ATCC 1705 and ATCC 700603, and the results are shown in the S2 Fig.

**Table 4:** The MIC of carbapenems for sRNA51-overexpressing strains of KPC-2-producing CRKP.

MIC	KPC-2 CRKP	KPC-2+sRNA51
Meropenem (μg/mL)	64	4
Ertapenem(μg/mL)	512	128
Imipenem (μg/mL)	> 1024	> 1024

### sRNA51 may affect KPC-2-producing CRKP resistance by regulating the expression of *acrA*

**Fig 4:**
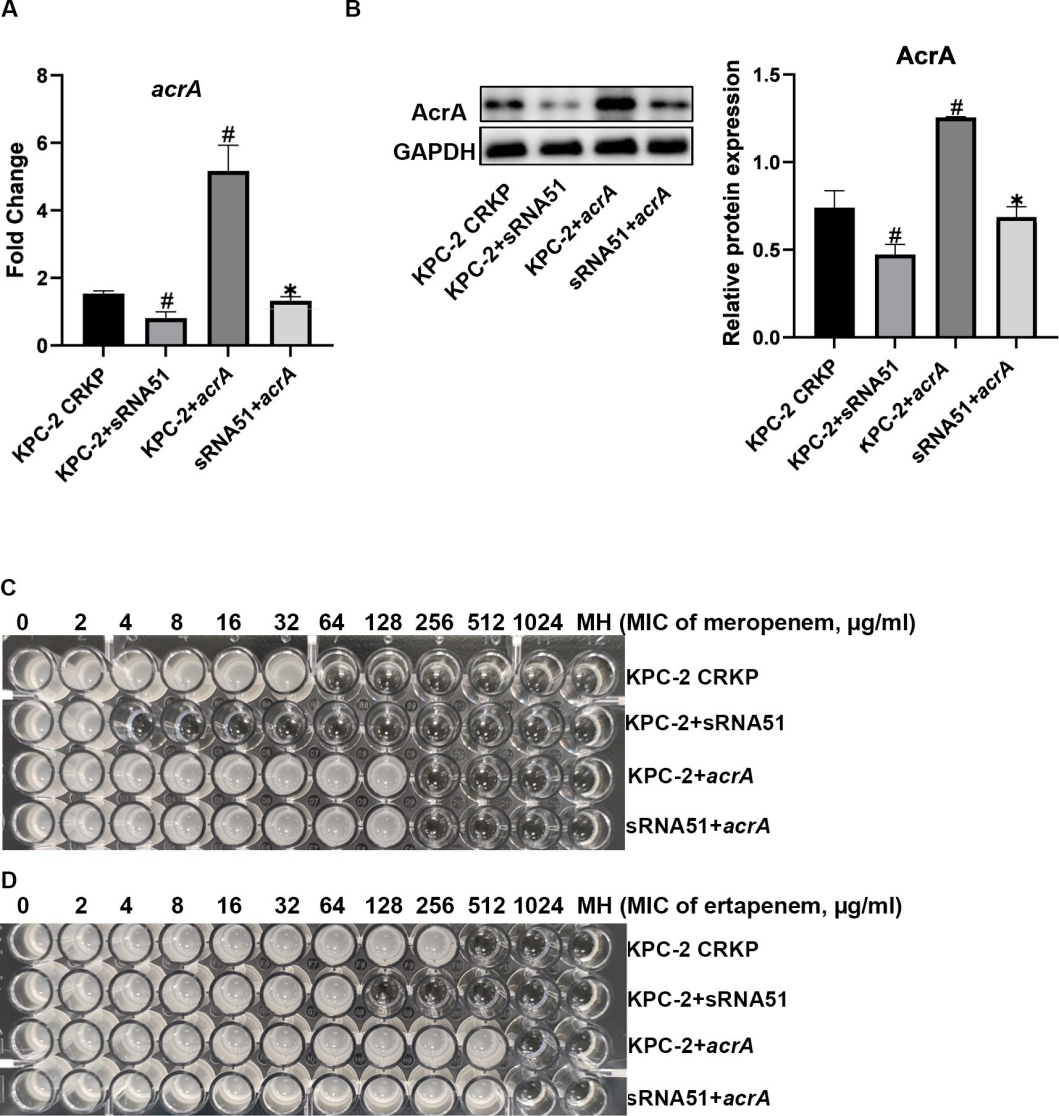
Mechanism of sRNA51 regulation carbapenems resistance of KPC-2-producing CRKP. qRT-PCR (A) and western blotting (B) detected expression levels of acrA in KPC-2+sRNA51 and sRNA51+*acrA* strains (n = 3). The data presented are representative images of three independent experiments with similar results (S3 Fig). Detection of resistance to meropenem (C) and ertapenem (D) in KPC-2+sRNA51 and sRNA51+*acrA* strains by microbroth dilution. Data are represented as the mean ± SD, ^#^ *P* < 0.05 indicates a significant difference compared with KPC-2-producing CRKP and *P < 0.05 indicates a significant difference compared with KPC-2+sRNA51.

To confirm above hypothesis, we overexpressed *acrA* in the KPC-2+sRNA51 strain (sRNA51+*acrA*). Following validation of successful construction by qRT-PCR and western blotting (Fig 4A and 4B), we analyzed the carbapenems resistance of each strain. The sRNA51+*acrA* strain exhibited increased resistance to meropenem to 256 μg/mL, and resistance to ertapenem increased to 1024 μg/mL (Fig 4C and 4D and Table 5). The above experiments were repeated using ATCC 1705, and the results are shown in the S2 Fig. Therefore, we conclude that sRNA51 can inhibit *acrA* expression and that the reduction of sRNA51 expression in KPC-2-producing CRKP can lead to an increase in *acrA* expression and carbapenems resistance by increasing the formation of efflux pumps.

**Table 5:** The MIC of carbapenems for overexpressed *acrA* in KPC-2+sRNA51 strains.

MIC	KPC-2 CRKP	KPC-2+sRNA51	KPC-2+*acrA*	sRNA51+*acrA*
Meropenem (μg/mL)	64	4	256	256
Ertapenem (μg/mL)	512	128	1024	1024

## Discussion

Currently, sRNA-mediated regulation is considered an RNA-based drug target against antibiotic-resistant bacteria [35]. sRNAs, which play a central role in post-transcriptional regulation by binding to their target mRNAs, are involved in several biological processes in bacteria, including regulation of virulence, stress response, metabolism, and antibiotic resistance [35,36]. In the initial few years, research on sRNAs mainly focused on *E*. *coli* and *Salmonella* strains [37]. In recent years, however, with the development of sequencing technology, RNA-seq has identified sRNAs in large numbers bacteria, many of which are capable of regulating more than one target mRNA, revealing a complex sRNA-based network [38,39]. However, the sRNA expression profile of KPC-2-producing CRKP and the role of sRNA-regulated mRNA combinations in KPC-2-producing CRKP antibiotic resistance remain unknown. In this study, we determined the sRNA expression profile using RNA-seq and identified the role of sRNA in KPC-2-producing CRKP.

Referring to methods described in previous studies [32,40], we first compared the available reads obtained from RNA-seq with a reference genome to obtain known genes using Bowtie2 (version 2.2.8). RNA-seq has been widely used in bacterial transcriptome sequencing owing to its ability for comprehensive and in-depth mining of transcriptome information and functionality [38]. Several studies have used RNA-seq to analyze differentially expressed RNA in drug-resistant strains to understand the mechanisms of antibiotic resistance [41,42]. We previously analyzed the transcriptome of *Streptococcus sanguis* SK36 and its CcpA-null derivative (△CcpA) using RNA-seq and obtained DEGs of △CcpA, which identified that CcpA is mainly involved in carbon catabolism and some amino acid catabolic pathways [43]. In this study, 4693 known genes were obtained from CSKP and KPC-2-producing CRKP isolates from clinical specimens. Of these, 307 genes were significantly differentially expressed in KPC-2-producing CRKP compared to CSKP, including 133 up-regulated and 174 down-regulated genes (Fig 1A and S3 Table). KEGG pathway enrichment and GO analysis revealed that these DEGs were mainly involved in metabolic processes, such as amino acid metabolism (Fig 1). Metabolism plays an important role in bacterial growth, drug resistance, and pathogenesis [44]. Shan et al. found that amino acids, including serine, threonine, glutamine, and tryptophan, could affect the sensitivity of *E*. *coli* to gentamicin [45]. Therefore, the role of metabolism in KPC-2-producing CRKP is worth exploring and will be the focus of our future research.

Unmatched genes were annotated using Rockhopper with the NR database, and we obtained 115 candidate sRNAs in CSKP and KPC-2-producing CRKP based on the following characteristics described previously [31,32]: (1) cannot be annotated with the NR database, (2) 50–500nt in length, and (3) stable secondary structure. sRNAMAP databse (genomic maps for sRNAs of all bacteria) and Rfam databse (a database of noncoding RNAs) are often used to annotate known sRNAs in bacteria [46,47]. Thus, we also used the sRNAMap database (version 2009) and the Rfam database (version 13) to match known sRNAs and found that only three sRNAs were known (*CsrC*, *CsrB* and *GlmY*) and 112 were newly discovered. Numerous similar studies have identified a substantial number of new sRNAs in bacteria. Krieger et al. identified 15 novel sRNAs in *Streptococcus mutants* and showed that they respond to four stress-inducing conditions commonly encountered by the pathogen in the human mouth [48]. Another study identified 71 new sRNAs that were differentially expressed in *Salmonella* in response to desiccation and demonstrated that knocking out sRNA1320429 or sRNA3981754 significantly impaired the ability of *Salmonella* to survive desiccation [49]. Houserova et al. identified 475 novel sRNAs in carbon-starved *Salmonella enterica* and demonstrated that the knockdown of sRNA4130247 significantly impaired the carbon starvation response of *salmonella* [50].

qRT-PCR is the primary tool used to verify the expression and characterization of novel sRNAs [51]. Therefore, we selected sRNA51, which was the most significantly differentially expressed sRNA in KPC-2-producing CRKP, as well as four other randomly selected differentially expressed sRNAs (sRNA93, sRNA156, sRNA398, and sRNA418), to examine the presence and transcript levels of sRNAs by qRT-PCR. The qRT-PCR results were consistent with those of RNA-seq, revealing that the data obtained by RNA-seq were efficient and reliable. And we selected sRNA51 for further analysis.

sRNA play the regulatory role mainly by regulating the expression of mRNA. Jia et al. found a novel sRNA EsrF that directly binds to the flagellar biosynthetic gene *flhB* to regulate its expression, thereby altering the motility and virulence of enterohemorrhagic *E*. *coli O157*: *H7* [52]. Study have found that sRNA PrrH negatively regulates *katA* to reduce the oxidative tolerance of *Pseudomonas aeruginosa* [53]. To further understand the role of sRNA51 in KPC-2-producing CRKP, we used IntaRNA 2.0.3 to predict its target genes, including *yaiY*, *gabT*, *msrA*, *acrA*, *insA1*, and *holE*. Among these, *acrA* attracted our attention. AcrAB-TolC is one of the most widespread multidrug efflux pumps in resistant gram-negative bacteria, through which bacterial expel antibiotics from their cells [33,54]. Fluoroquinolone and tigecycline resistance in *K*. *pneumoniae* is attributed to the overexpression of *ramA* and subsequent upregulation of *acrA* [55,56]. Additionally, study have indicated that sRNA CsrA positively regulates acrA levels by stabilizing *acrA* mRNA [57]. However, studies on *acrA* and CRKP have primarily focused on the relationship between *acrA* expression and carbapenems resistance. For instance, *acrA* expression levels were found to be elevated in CRKP isolates, and reducing *acrA* expression can improve carbapenems sensitivity in CRKP [28,34]. The mechanism underlying the regulation of *acrA* expression in CRKP remains unknown. Consistent with the results of other studies, we found that the RNA and protein expression levels of acrA in KPC-2-producing CRKP were higher than those in CSKP.

To demonstrate the association between sRNA51 and *acrA* in KPC-2-producing CRKP, we constructed sRNA51-overexpressing KPC-2-producing CRKP strains and found that, compared with KPC-2-producing CRKP, overexpression of sRNA51 reduced the expression of *acrA*, and reduced its resistance of meropenem from 64 μg/mL to 4 μg/mL, resistance of ertapenem decreased from 512 μg/mL to 128 μg/mL (Fig 3). We then performed a rescue experiment to overexpress *acrA* in the sRNA51-overexpressing strains. The results showed that *acrA* overexpression restored its resistance of meropenem and ertapenem in sRNA51-overexpressing strains to 256 μg/mL and 1024 μg/mL respectively (Fig 4). Therefore, we concluded that sRNA51 can inhibit the expression of *acrA* and that down-regulation of sRNA51 in KPC-2-producing CRKP leads to increased expression of *acrA* and increases its resistance through efflux pumps.

In this study, we showed the sRNA expression profiles of KPC-2-producing CRKP, as well as differentially expressed sRNAs compared to CSKP. Moreover, the study of sRNA51 provided new insights into the interaction between *acrA* and carbapenems resistance in KPC-2-producing CRKP, enriched our understanding of the mechanism of carbapenems resistance in KPC-2-producing CRKP, and provided a possible target for RNA therapy of KPC-2-producing CRKP.

## Materials and methods

### Bacterial strains

Over the past decade, Shengjing Hospital, affiliated with China Medical University has established a pathogen bank isolated from clinical specimens, including a variety of drug-resistant and drug-sensitive fungi and bacteria. Between 2019 to 2021, 153 CRKP strains were collected, including 121 KPC-2-producing CRKP strains. Ten strains of KPC-2-producing CRKP from patients with sepsis and 10 strains of CSKP from patients with pneumonia were selected for the follow-up experiments using the SPSS random sampling method. All strains were ST11. *Klebsiella pneumoniae* ATCC 1705 and *Klebsiella pneumoniae* ATCC 700603 were used as the standard strains for KPC-2-producing CRKP and CSKP, respectively.

### Antibiotic sensitivity test

The Phoneix 100 (BD) compact system was used to detect the resistance of selected strains, and the results were interpreted according to the Clinical and Laboratory Standards Association.

The microbroth dilution method was based on previous studies with some modifications [58]. Briefly, the bacterial solution was cultured to a concentration of 5×10^7^ cfu/mL and diluted 100 times. Meropenem was diluted to 2048, 1024, 512, 256, 128, 64, 32, 16, 8, and 4 μg/mL in MH medium. Subsequently, 50 μL of the antibiotic was added to the corresponding well of the 96-well plate, followed by the addition of 50 μL of diluted bacterial solution to each well. The resulting meropenem concentrations were 1024, 512, 256, 128, 64, 32, 16, 8, 4, and 2 μg/mL, while the final bacterial density was 5×10^5^ cfu/mL. Negative (MH medium only) and positive control wells (no drugs) were established simultaneously. After 16–18 h of culture, colony growth was observed with the naked eye. The drug concentration without bacterial growth was regarded as the minimum inhibitory concentration. Microbroth dilution methods for imipenem and ertapenem were identical to those described above. According to the Clinical and Laboratory Standards Institute (CLSI) criteria, *K*. *pneumoniae* is resistant to meropenem and imipenem at MIC ≥ 4 μg/mL and resistant to ertapenem at MIC ≥ 2 μg/mL.

### Extraction of RNA

KPC-2-producing CRKP and CSKP strains were inoculated in 10 mL LB medium and cultured overnight (37°C, 200 rpm). The organisms were obtained after centrifugation (1000 g, 3 min). Total RNA was isolated using the SPINeasy RNA Kit for Bacteria (with Lysing Matrix) (MP), and its concentration and purity were estimated using K5600 (260 nm/280 nm, KaiAo) and separated on 2% agarose gels (S4 Fig).

### RNA and sRNA Sequencing

Five strains of KPC-2-producing CRKP and five strains of CSKP were used for RNA-seq (Gene Denovo Biotechnology). Illumina NovaSeq 6000 sequencing and FASTP (https://github.com/opengene/fastp) software were used to obtain valid data.

The resulting data were compared with the reference genome assembly GCF_022869665.1 (https://www.ncbi.nlm.nih.gov/assembly/12472981) to obtain known genes by using Bowtie2 (version 2.2.8).

According to previous studies [31,32], we annotated the unmatched genes with the NR database using Rockhopper. Unannotated genes with 50–500 nt of length and stable secondary structures were listed as candidate sRNAs [46]. In addition, we used the sRNAMap database (version 2009) and the Rfam database (version 13) to match known sRNAs.

### DEGs and differentially expressed sRNA analysis

RSEM software and fragments per kilobase of transcript per million fragments mapped (FPKM) were used to calculate the expression levels of RNAs and sRNAs [59]. In addition, EdgeR software was used to screen DEGs and differentially expressed sRNAs with the criteria of FDR < 0.05 and | log2FC | > 1.

### Prediction of ORF and target mRNA of sRNA

It is well-known that all mRNAs with protein-coding functions have ORFs; sRNAs do not have ORFs but are intergenic segments located between ORFs. Therefore, CPAT software was used to assess whether a gene has an ORF to code for proteins [60]. As per the literature, the criteria are as follows: Fickett score ≤ 0.74 indicates a lack of coding ability, Fickett score ≥ 0.95 indicates coding ability, and Fickett score between 0.74 and 0.95 indicates uncertainty about the coding. In addition, a positive Hexamer score signifies coding potential. The larger the value, the more likely it encodes proteins [61,62].

The target mRNA of the sRNA and the binding sites of the sRNA to the mRNA were predicted using IntaRNA 2.0.3 and drawn using FigDraw.

### KEGG pathway and GO analyses

KEGG pathway enrichment analysis uses a hypergeometric detection method to enrich DEGs in the pathway. GO analysis maps all differential genes to each term in the GO database, including molecular function, biological process, and cellular components, and counts the number of genes in each term.

### qRT-PCR

The primers used for sRNA51, sRNA93, sRNA156, sRNA398, sRNA418, and *acrA* are shown in Table 6. The cDNA was prepared using a reverse transcription kit (Vazyme), with 2 μL cDNA as a template according to the instructions of the 2×ChanQ Universal SYBR qPCR Master kit (Vazyme). Using the *rpoB* gene as the reference gene, the expression of the relative target gene was calculated by the 2^−ΔΔCt^ method.

**Table 6:** Primer sequences used for qRT-PCR.

Gene	Forward primer (5′-3′)	Reverse primer (5’-3′)
sRNA156	CATGCGGAAGCGAAGTACGTTC	CGAAGTGTGTCACCTGGGAGAG
sRNA51	GAGATGCGCCATAATTAAGGTC	CCTACGATGGTGAAAGAGTGGTG
sRNA398	CGCTTACCAACAATACGGATCTGG	ATCTGATGAGCGCCGCTTCG
sRNA93	CATACCCGTCATATTTCCGTAGCC	CTACCCTGAATCATTCACCTTGCC
sRNA418	GGCGGGACAACGATTATAAAGAGC	ACGACCGAGTACACGGAATGTC
*rpoB*	AAGGCGAATCCAGCTTGTTCAGCC	TGACGTTGCATGTTCGCACCCATCA
*acrA*	CAGGAAAACGGCAAAGCGAA	ATAGCGCGTAGCGTGATTGA

### Western blotting

Western blotting was performed as previously described, with some modifications [57]. Overnight cultures of *K*. *pneumoniae* were centrifuged and resuspended in 1 mL 50 mM Tris/HCl (pH 8.0) and ultrasonically lysed on ice (four 30-second pulses, with a 30-second pause between each pulse). The Omni-Easy Instant BCA Protein Assay Kit (EpiZyme) was used to quantify protein concentration. Proteins were electrophoresed in Tris-glycine-sodium dodecyl sulfate electrophoresis buffer (EpiZyme). Separated proteins were transferred to PVDF membranes in Omni-Flash transmembrane buffer (EpiZyme). After washing, the membranes were incubated with antibodies against AcrA (custom-made by Abmart) and GAPDH (GeneTex) overnight (GAPDH was used as an internal reference). The membranes were washed and incubated with an enzyme-linked rabbit secondary antibody (Absin). Protein bands were captured using an enhanced chemiluminescence (ECL, EpiZyme) western blotting detection system (Tanon) and densitometry was performed using Tanon Image software (Tanon).

### Construction of KPC-2+sRNA51 and sRNA51+*acrA* strain

We commissioned KMD Biosciences to construct a plasmid containing a kanamycin resistance gene. The KPC-2+sRNA51 strain was constructed as follows: sRNA51 was amplified using the forward primer CAGCGGCCTGGTGCCGCGCGGCAGCCATATATGCCATAAACAGTTGCGTCGGGCCGCGTGGGC and reverse primer GATCTCAGTGGTGGTGGTGGTGGTGCTCGAGGCTGTATGGTGGGGTCAGGGGGTTGGGGT. Then sRNA51 was cloned into the pET-28a (+) plasmid which carried the kanamycin resistance gene, and electroporated into *E*. *coli* S17-1λpir. Recipient KPC-2-producing CRKP and donor *E*. *coli* were separately cultured to 5×10^7^ cfu/mL, mixed at a ratio of 2:1 (donor: recipient), and inoculated on LB agar plates overnight (37°C). The mixture was resuspended in 1 mL LB and 100 μL was inoculated into LB agar plates containing kanamycin overnight (37°C) to screen mutants [63]. If the plasmid was successfully grafted onto KPC-2-producing CRKP, colonies grew on the plates.

The sRNA51+*acrA* overexpression strain was constructed in the same manner as the sRNA51- overexpression strain. The primers of *acrA*: forward primer CAGCGGCCTGGTGCCGCGCGGCAGCCATATGAACAAAAACAGAGGGTTAACGCCTCTGGCGG and reverse primer GATCTCAGTGGTGGTGGTGGTGCTCGAGGGGGAAGATAGCGCGTAGGGTGATAGACCCAGTGGTC. The recipient strain was sRNA51- overexpression strain.

### Statistical analysis

Statistical was calculated using GraphPad Prism 9.0.0. Firstly, Shapiro-Wilk method was used to check whether the calculated data fit the normal distribution. If no, the Mann-Whitney test is used; if yes, the variance is tested; if the variance is consistent, the unpaired t test is used; if no, Welch’s correction is used. Differences were considered statistically significant at *P <* 0.05.

## Supporting information

S1 Table The information of CRKP strains in pathogen bank of Shengjing Hospital, affiliated with China Medical University. (XLSX)

S2 Table Information of *Klebsiella pneumoniae* used in this study. (XLSX)

S3 Table DEGs in KPC-2-producing CRKP compared to CSKP. (XLSX)

S4 Table KEGG pathway enrichment of DEGs. (XLSX)

S5 Table GO analysis of DEGs. (XLSX)

S6 Table The sRNA expression profile of KPC-2 CRKP and CSKP. (XLSX)

S7 Table Antibiotic resistance and informatiomn of *Klebsiella pneumoniae* used in this study. (XLSX)

S8 Table Target mRNAs of sRNA51. (XLSX)

S9 Table Raw data obtained in this study. (XLSX)

S1 Fig The expression levels of sRNAs in ATCC 1705 and ATCC 700603. The expressions of sRNA51 (A), sRNA93 (B), sRNA156 (C), sRNA398 (D), and sRNA418 (E) in ATCC 1705 and ATCC 700603 were detected by qRT-PCR. Data are expressed as mean± SD (n = 3), * *P* < 0.05 and ** *P* < 0.01.
(TIF)

S2 Fig The role of sRNA51 in ATCC 1705. The RNA (A) and protein (B) expression level of acrA in ATCC 1705 and ATCC 700603 were detected by qRT-PCR and western blotting (n = 3). Data are represented as the mean±SD, ** *P* < 0.01 and *** *P* < 0.001. The RNA expression levels of sRNA51 (C) and *acrA* (D) in ATCC 1705 and 1705+sRNA51 strains were detected by qRT-PCR (n = 3).Data are represented as the mean±SD,**P < 0.01. (E) Western blotting analysis of the protein expression level of acrA in ATCC 1705 and 1705+sRNA51 strains. Data are represented as the mean±SD, *P < 0.05. (F) Resistance of ATCC 1705 and 1705+sRNA51 strains to meropenem, ertapenem and imipenem was detected by microbroth dilution. qRT-PCR (G) and western blotting (H) detected expression levels of acrA in 1705+*acrA* and sRNA51+*acrA* strains (n = 3). Data are represented as the mean±SD, ^#^*P* < 0.05, ^##^*P* < 0.01 and ^###^*P* < 0.0001 indicates a significant difference compared with ATCC 1705, **P < 0.01 and ***P < 0.001 indicates a significant difference compared with 1705+sRNA51.(I) Detection of resistance to meropenem and ertapenem in 1705+ *acrA* and sRNA51+*acrA* strains by microbroth dilution.
(TIF)

S3 Fig Western blotting images of AcrA. (A) Western blotting detected expression levels of acrA in KPC-2-producing CRKP and KPC-2+sRNA51 strains (n = 3). (B) Western blotting detected expression levels of acrA in KPC-2+*acrA* and sRNA51+*acrA* strains (n = 3).
(TIF)

S4 Fig Gels of RNA. (A) RNA agarose gel electrophoresis of CRKP and CSKP. (B) RNA agarose gel electrophoresis of KPC-2-producing CRKP, KPC-2+sRNA51, KPC-2+*acrA* and sRNA51+*acrA* strains. (C) RNA agarose gel electrophoresis of ATCC 700603, ATCC 1705, 1705+sRNA51, 1705+*acrA* and sRNA51+*acrA* strains.
(TIF)
