# Supplementary figures and images for: sRNA expression profile of KPC-2-producing carbapenem-resistant Klebsiella pneumoniae: Functional role of sRNA51
Source: PLoS Pathog. 2024 May 8;20(5):e1012187. doi: 10.1371/journal.ppat.1012187 (PMC11078416; doi:10.1371/journal.ppat.1012187)

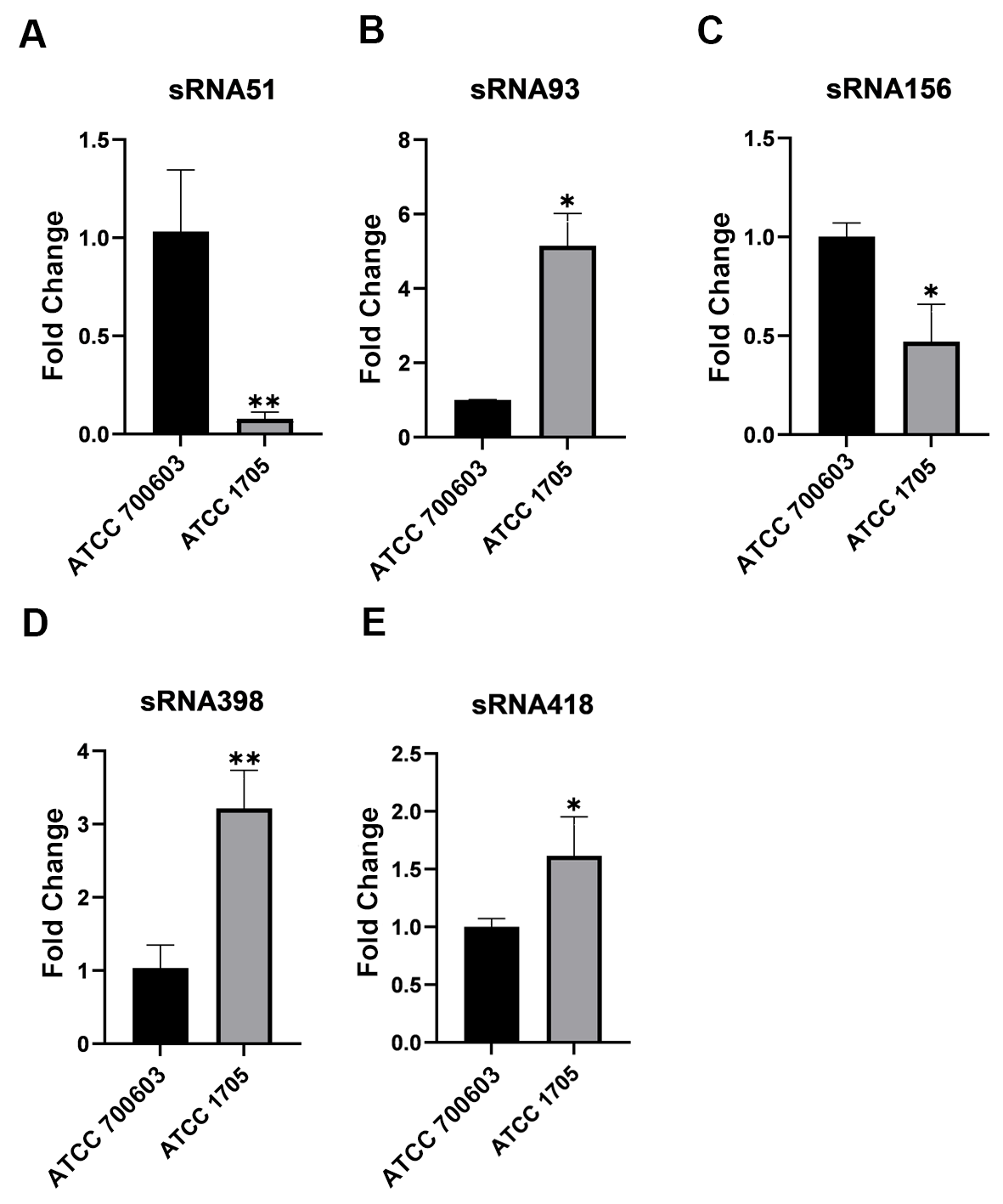

Supplement: S1 Fig — The expressions of sRNA51 (A), sRNA93 (B), sRNA156 (C), sRNA398 (D), and sRNA418 (E) in ATCC 1705 and ATCC 700603 were detected by qRT-PCR. Data are expressed as mean± SD (n = 3), * P < 0.05 and ** P < 0.01. (TIF) [file ppat.1012187.s010.tif]

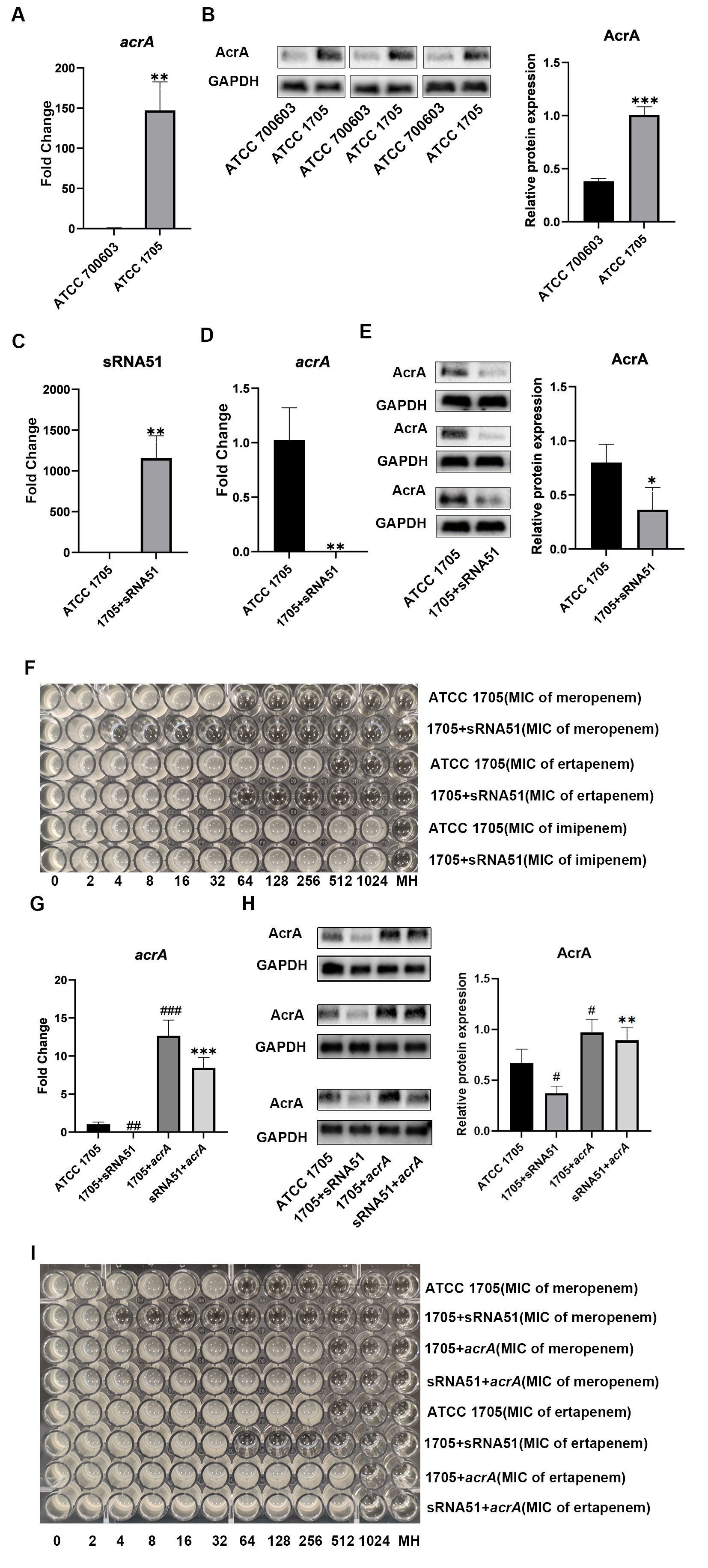

Supplement: S2 Fig — The RNA (A) and protein (B) expression level of acrA in ATCC 1705 and ATCC 700603 were detected by qRT-PCR and western blotting (n = 3). Data are represented as the mean±SD, ** P < 0.01 and *** P < 0.001. The RNA expression levels of sRNA51 (C) and acrA (D) in ATCC 1705 and 1705+sRNA51 strains were detected by qRT-PCR (n = 3).Data are represented as the mean±SD,**P < 0.01. (E) Western blotting analysis of the protein expression level of acrA in ATCC 1705 and 1705+sRNA51 strains. Data are represented as the mean±SD, *P < 0.05. (F) Resistance of ATCC 1705 and 1705+sRNA51 strains to meropenem, ertapenem and imipenem was detected by microbroth dilution. qRT-PCR (G) and western blotting (H) detected expression levels of acrA in 1705+acrA and sRNA51+acrA strains (n = 3). Data are represented as the mean±SD, #P < 0.05, ##P < 0.01 and ###P < 0.0001 indicates a significant difference compared with ATCC 1705, **P < 0.01 and ***P < 0.001 indicates a significant difference compared with 1705+sRNA51.(I) Detection of resistance to meropenem and ertapenem in 1705+ acrA and sRNA51+acrA strains by microbroth dilution. (TIF) [file ppat.1012187.s011.tif]

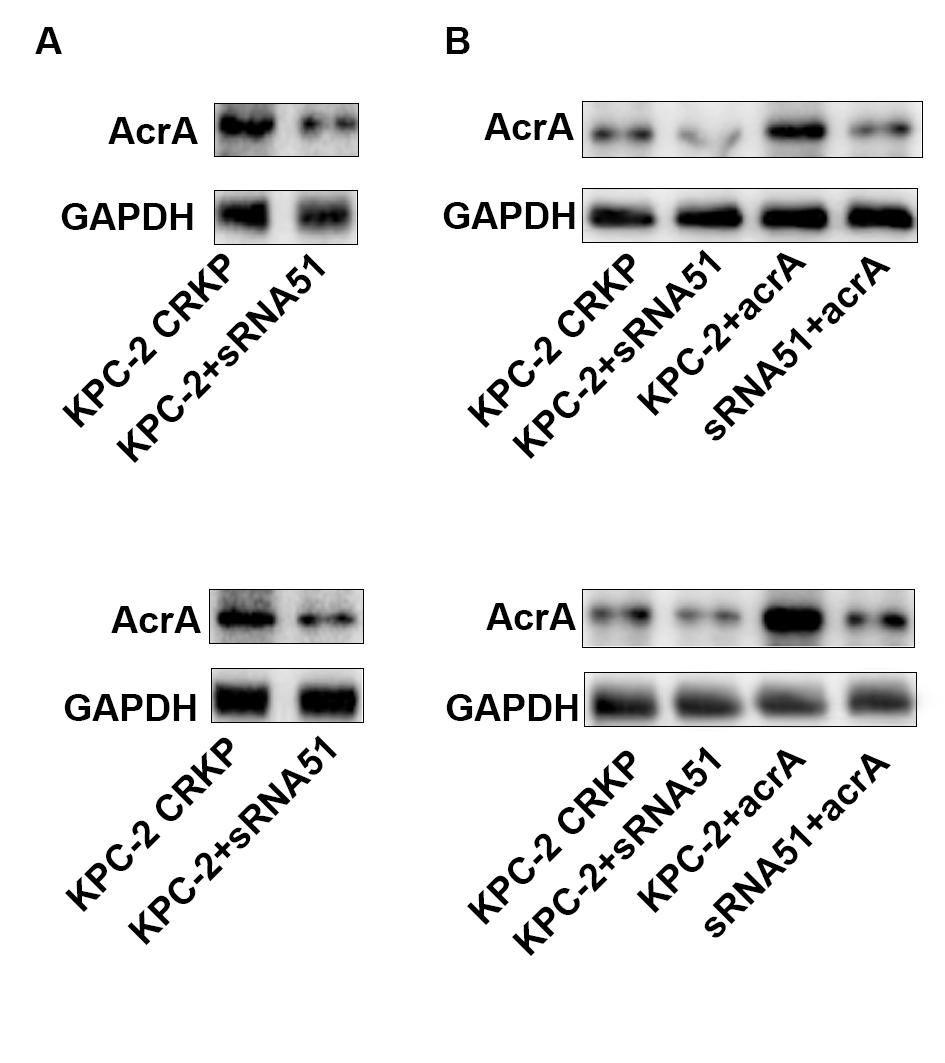

Supplement: S3 Fig — (A) Western blotting detected expression levels of acrA in KPC-2-producing CRKP and KPC-2+sRNA51 strains (n = 3). (B) Western blotting detected expression levels of acrA in KPC-2+acrA and sRNA51+acrA strains (n = 3). (TIF) [file ppat.1012187.s012.tif]

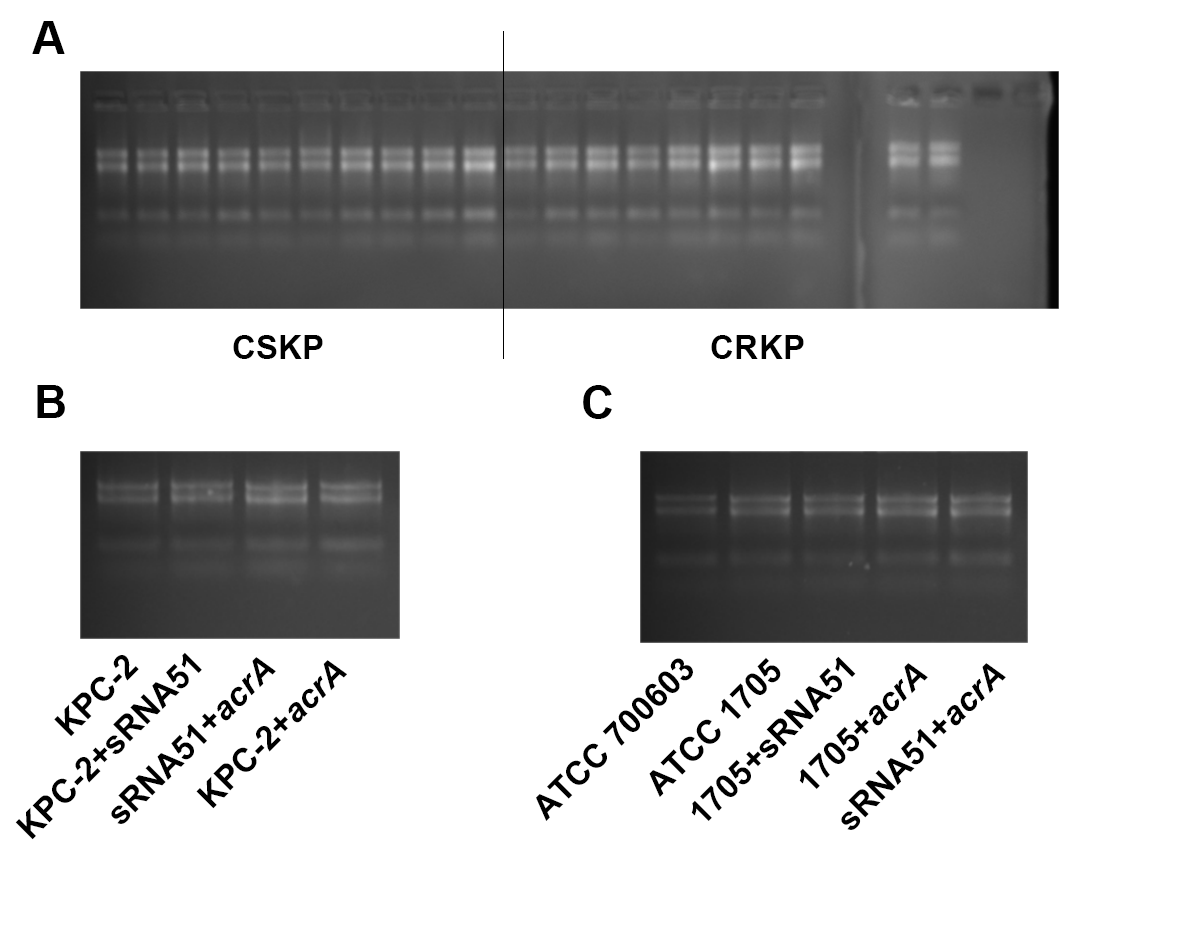

Supplement: S4 Fig — (A) RNA agarose gel electrophoresis of CRKP and CSKP. (B) RNA agarose gel electrophoresis of KPC-2-producing CRKP, KPC-2+sRNA51, KPC-2+acrA and sRNA51+acrA strains. (C) RNA agarose gel electrophoresis of ATCC 700603, ATCC 1705, 1705+sRNA51, 1705+acrA and sRNA51+acrA strains. (TIF) [file ppat.1012187.s013.tif]
